# Non-thermal structural transformation of diamond driven by x-rays

**DOI:** 10.1063/4.0000193

**Published:** 2023-10-27

**Authors:** Philip Heimann, Nicholas J. Hartley, Ichiro Inoue, Victor Tkachenko, Andre Antoine, Fabien Dorchies, Roger Falcone, Jérôme Gaudin, Hauke Höppner, Yuichi Inubushi, Konrad J. Kapcia, Hae Ja Lee, Vladimir Lipp, Paloma Martinez, Nikita Medvedev, Franz Tavella, Sven Toleikis, Makina Yabashi, Toshinori Yabuuchi, Jumpei Yamada, Beata Ziaja

**Affiliations:** 1 Linac Coherent Light Source, SLAC National Accelerator Laboratory, 2575 Sand Hill Road, Menlo Park, California 94025, USA; 2 SLAC National Accelerator Laboratory, 2575 Sand Hill Road, Menlo Park, California 94025, USA; 3 RIKEN SPring-8 Center, 1-1-1 Kouto, Sayo, Hyogo 679-5148, Japan; 4 European XFEL GmbH, Holzkoppel 4, 22869 Schenefeld, Germany; 5 Center for Free-Electron Laser Science CFEL, Deutsches Elektronen-Synchrotron DESY, Notkestr. 85, 22607 Hamburg, Germany; 6 University of Michigan, 500 S State St, Ann Arbor, Michigan 48109, USA; 7 University Bordeaux, CNRS, CEA, CELIA, UMR 5107, F-33500 Talence, France; 8 Department of Physics, University of California, Berkeley, California 94720, USA; 9 Helmholtz-Zentrum Dresden-Rossendorf, 01328 Dresden, Germany; 10 Japan Synchrotron Radiation Research Institute, 1-1-1 Kouto, Sayo, Hyogo 679-5198, Japan; 11 Institute of Spintronics and Quantum Information, Faculty of Physics, Adam Mickiewicz University in Poznań, ul. Uniwersytetu Poznańskiego 2, 61-614 Poznań, Poland; 12 Institute of Nuclear Physics, Polish Academy of Sciences, Radzikowskiego 152, 31-342 Krakow, Poland; 13 Institute of Physics, Czech Academy of Sciences, Na Slovance 1999/2, 182 21 Prague 8, Czech Republic; 14 Institute of Plasma Physics, Czech Academy of Sciences, Za Slovankou 3, 182 00 Prague 8, Czech Republic; 15 Deutsches Elektronen-Synchrotron DESY, Notkestr. 85, 22607 Hamburg, Germany

## Abstract

Intense x-ray pulses can cause the non-thermal structural transformation of diamond. At the SACLA XFEL facility, pump x-ray pulses triggered this phase transition, and probe x-ray pulses produced diffraction patterns. Time delays were observed from 0 to 250 fs, and the x-ray dose varied from 0.9 to 8.0 eV/atom. The intensity of the (111), (220), and (311) diffraction peaks decreased with time, indicating a disordering of the crystal lattice. From a Debye–Waller analysis, the rms atomic displacements perpendicular to the (111) planes were observed to be significantly larger than those perpendicular to the (220) or (311) planes. At a long time delay of 33 ms, graphite (002) diffraction indicates that graphitization did occur above a threshold dose of 1.2 eV/atom. These experimental results are in qualitative agreement with XTANT+ simulations using a hybrid model based on density-functional tight-binding molecular dynamics.

## I. INTRODUCTION

In metals, thermal melting induced by short pulses of light occurs on picosecond time scales, triggered by electron–phonon coupling.^1^ In covalently bonded materials, exposure to a short burst of either x-ray or optical radiation^2^ may induce a non-thermal phase transition. For a non-thermal phase transition, calculations predict that the excitation of a few percent of the valence band electrons leads to a modification of the potential energy surface, which results in a change in the atomic structure on femtosecond timescales.^3,4^ With x-ray pump pulses focused on diamond, previous work has observed a non-thermal structural disordering^5^ and a transition to another crystalline phase, graphite, at lower pump intensities.^6,7^ For the solid-to-solid transformation, there is indirect evidence that the phase transition is non-thermal from the measurement of the optical transmission.^7^ Calculations have also predicted the x-ray-induced phase transition from diamond to graphite^4^ although more recent simulations indicate partial graphitization.^8^

Reitze *et al.* conducted optical pump-optical probe measurements of graphite and diamond and derived optical properties for liquid carbon.^9^ In comparison with the optical pump-optical probe technique, the x-ray pump-probe method involves a 'simpler' interaction of the x-rays with materials (with photoabsorption being the only channel for the absorption of the x-ray energy) and an uniform absorption with depth (in contrast to the absorption of optical radiation, which is strongly non-uniform with depth).^10^ High energies of the probing x-ray photons can provide very high spatial resolution for the structural changes observed.^11^ On the other hand, with the x-ray pump-probe measurements, there is a spatial gradient of pulse intensity and a spatial transport of the energetic photo and Auger electrons. The latter can significantly decrease the energy deposition in the beam focus. Both these effects imply that the observed diffraction signal is volume integrated, i.e., it collects the contributions from differently illuminated regions of the material. This makes a comparison between experimental and theory predictions not straightforward, as the latter should also include volume integration of the predicted diffraction signal. Gaudin *et al.*^6^ examined the threshold fluence of graphitization in irradiated diamond for a variety of extreme ultraviolet wavelengths and demonstrated from *ex situ* Raman measurements that a phase transition does occur. Using soft x-ray irradiation, Tavella *et al.*^7^ observed the graphitization phase transition indirectly through changes in the optical transmission.

The interaction of diamond with high-intensity x-rays has applications in many areas of high energy density physics. One such area of interest is found in inertial confinement fusion (ICF); recent experiments performed at the National Ignition Facility have achieved a burning plasma state using nanocrystalline diamond as the ablator material.^12^ Also, many devices used in x-ray free-electron laser (FEL) beamlines involve diamond and silicon.^13–15^ Thus, a good understanding of the damage mechanisms within these materials is of great practical utility for the operation of x-ray FELs. These studies can give further prospects for the ultrafast processing of materials with intense x-ray pulses.^16^ In general, the elucidation of diamond's response to x-ray radiation will provide us with fundamental knowledge related to how changes in a material's electronic structure can affect its atomic structure and, hence, provide new insight into several aspects of high energy density physics and materials science.

Under femtosecond x-ray irradiation, covalently bonded materials, such as diamond, undergo a sequence of processes.^17,18^ First, photoabsorption promotes electrons from the bound states of the deep atomic shells (K-shell for carbon) or valence band to the conduction band. The deep-shell holes can then decay through Auger processes, which are the predominant relaxation channels for low-Z (light) elements.^4^ The Auger decay results in more electronic excitations from the valence band to the conduction band, following the relaxation of the core holes into the valence band. The released photo- and Auger electrons scatter further via inelastic (impact ionization of valence band or deep-shell electrons) and elastic channels. In the case of 10 keV photoelectrons, the impact ionization cascading is completed on a 100-femtosecond timescale. It finishes when the electrons do not have sufficient energy to perform more impact ionizations.^19^ Meanwhile, initiated by 10 keV photoelectrons, the spatial distribution of the secondary electron cloud expand to 600 nm.^20^

Ziaja and co-workers have developed a theoretical framework for modeling x-ray-induced phase transitions using hybrid models. The resulting simulation tools are (i) the XTANT code based on tight-binding molecular dynamics^4,21,22^ and (ii) the XTANT+ code based on density-functional tight-binding molecular dynamics.^8,11^ These models include photoabsorption, Auger decay, and collisional scattering processes occurring in the electronic system.^4,8^ Because of these processes, the occupation of electronic bands transiently changes, causing the evolution of the potential energy surface. In diamond, populating a sufficient number of antibonding states could lead to an ultrafast rearrangement of atoms from sp^3^ to sp^2^ bonding, causing its transition to graphite-like structures. The present experiment provides a test of the accuracy of these theoretical methods. This can validate their applicability to obtain predictions on the x-ray interaction with materials. For the present experiment, we use the XTANT+ tool, first introduced in 2022.^8^

For the observation of graphitization, this experiment was performed at the SACLA x-ray free electron laser facility^23^ operating in the two-color mode.^24^ The first x-ray pulse triggered a phase transition. The second x-ray pulse probed the structural change through x-ray diffraction. The intensity of the diamond reflections was monitored with respect to the time separation between the two x-ray pulses. These experimental results are then compared with XTANT+ calculations.

## II. EXPERIMENTAL

The SACLA x-ray Free Electron Laser was operated in the split undulator mode generating x-ray pump and probe pulses.^24^ The two pulses consisted of pump pulses at a photon energy of 7 keV and probe pulses at 10.5 keV, both ∼6 fs in duration.^25,26^ The jitter between the x-ray pump and probe pulses is less than 1 fs.^24^ The pump-probe time delay was varied between 0 and 250 fs. A diamond spectrometer measured the x-ray spectrum and, in particular, determined the pump and probe pulse energies.^27^ At the sample, the average pulse energies were 23 *μ*J for the pump pulses and 14 *μ*J for the probe pulses. The pump and probe pulse energies varied following the self-amplified spontaneous emission process. Events were sorted according to the pump pulse energy into four bins each with a width of ±3 *μ*J. At BL3 experimental hutch 5,^28^ the Kirkpatrick–Baez mirrors focused the x-rays to spot size of 140 × 160 nm (full-width at half-maximum). To vary the x-ray dose, the sample was translated along the x-ray propagation direction, which resulted in increased x-ray focal areas up to 1.06 × 1.05 *μ*m^2^. The average profile of both pump and probe beams was measured at a set of sample translations and shown to be two-dimensional Gaussians. The pump pulse was selected by kicking the electron beam at the magnetic chicane preventing emission of the probe pulse from the downstream undulators. The probe pulse was isolated with a 1.6-mm-thick silicon filter, attenuating the pump and probe by 9 × 10^−15^ and 5 × 10^−5^, respectively.^29^ The spatial separation between the pump and probe beams was observed to be ∼20 nm. By a continuous motion of the sample along a serpentine path, an undamaged location was provided for each x-ray pump-probe exposure of the sample. There were between 6000 and 25 000 exposures for each x-ray spot size and time delay, where the largest number of shots was performed with the smallest spot size and the smallest number with the largest spot size.

**FIG. 1.**
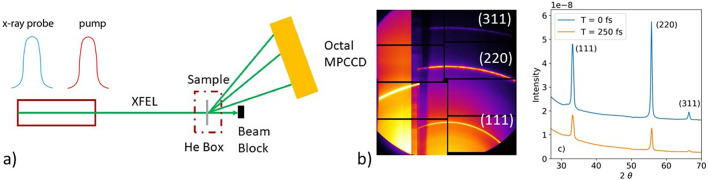
(a) The experimental setup at the SACLA experimental hutch 5, (b) a selected diffraction image, and (c) azimuthally integrated diffraction patterns at 0 and 250 fs time delay.

Figure 1 shows (a) the experimental setup, (b) a probe diffraction pattern on the Multi-Port Charge-Coupled Device (MPCCD) detector,^30^ and (c) azimuthally averaged diffraction patterns at 0 and 250 fs time delay and 3.1 eV/atom x-ray dose. The left side of the detector was covered by a 50 *μ*m thick Cu filter to select the pump diffraction and the right side by a 600 *μ*m thick Al filter to select the probe diffraction. The samples were 20 *μ*m thick nanocrystalline free-standing diamond (Diamond Materials). The thickness is more than an order of magnitude less than the x-ray attenuation length at the pump photon energy of 7 keV (428 *μ*m),^29^ implying that the x-rays are uniformly absorbed through the depth of the sample. From the Scherrer equation, the average nanocrystal size is estimated as 20 nm. From scanning electron microscope measurements, diamond films fabricated by the same method were analyzed to have an average grain size of 30 nm.^31^ The samples were inside a small helium enclosure. The nanocrystalline diamond produced a powder diffraction pattern as seen in Fig. 1(b). The octal MPCCD collected the (111), (220), and (311) diffraction peaks. From a comparison of the diffraction intensity distribution along the azimuthal angle, χ, with the polarization factor, it is concluded that the nanocrystal orientation is nearly isotropic. It is not expected that the dimensions of the nanocrystals would contribute dynamics on the 250 fs time range of this experiment; in nanocrystalline gold films, the propagation of melting from grain boundaries has been observed but with time scales of ∼50 ps.^32^

## III. RESULTS

**FIG. 2.**
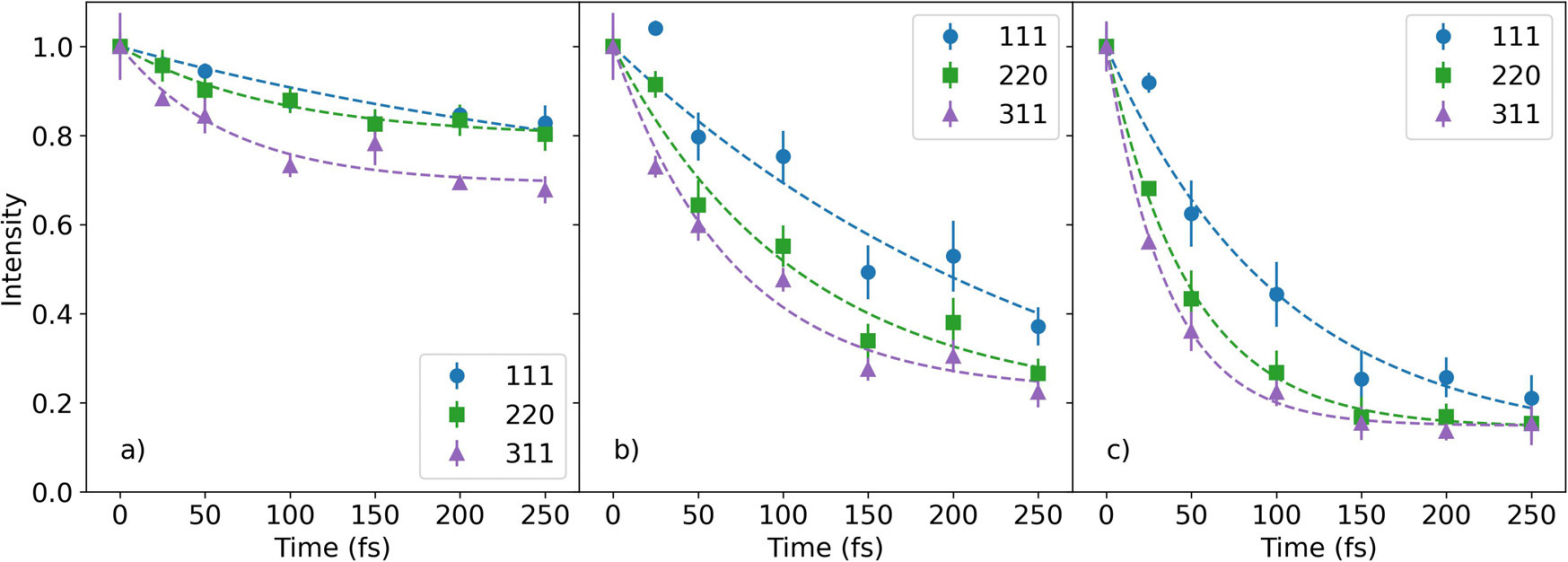
The time-dependent intensities of the (111), (220), and (311) diffraction peaks at the effective x-ray doses of (a) 0.9, (b) 3.1, and (c) 6.1 eV/atom. The curves are fits to an exponential function.

Three diffraction peaks are observed, and the diamond reflections are (111), (220), and (311). To extract the peak intensities, the peaks were fit using a Lorentzian function, $\frac{I}{\pi w(1+{\left(\frac{x-x_{c}}{w}\right)}^{2})}+B$, where *I* is the intensity, *w* is the half-width at half-maximum (HWHM), *x* is the scattering angle (2θ), and *B* is the background. The background is a linear function including a constant and slope. A first normalization was made to the probe pulse intensities observed by a spectrometer in an upstream hutch and then a second normalization to the diffraction intensity at the pump-probe time delay of 0 fs. Figure 2 shows the diffraction intensities between 0 and 250 fs at a low, medium, and high x-ray dose. The effective x-ray dose *D*_eff_ is calculated as follows:

$$D_{\text{eff}}=\frac{E}{\sqrt{w_{x}^{2}+\lambda_{e}^{2}}\sqrt{w_{y}^{2}+\lambda_{e}^{2}}\lambda_{x}\rho_{a}},$$ (1)

where E is the pump pulse energy, w_x_ is the pump horizontal x-ray focus (width at the 1/e level), w_y_ is the pump vertical x-ray focus (width at the 1/e level), λ_e_ is the electron cascade size at the pump photon energy of 7 keV (0.41 *μ*m according to Refs. 20 and 33), λ_x_ is the x-ray penetration depth at the pump photon energy, and ρ_a_ is the atomic density. The pump pulse energy is evaluated from the pump pulse intensity observed by the spectrometer, which is then normalized by the signal of a beamline intensity monitor and corrected by the reflectivity of the Kirkpatrick–Baez focusing mirrors. The inclusion of λ_e_ in Eq. (1) represents an approximation because λ_e_ is a maximum distance at which the electrons lose their energy below a cutoff.^20^ The electron cascade size increases the volume of energy deposition especially for the smaller x-ray spot sizes, i.e., higher effective doses. In addition, the pump and probe beams have Gaussian intensity profiles, which results in different regions of the sample being excited by a range of x-ray doses. Therefore, one cannot expect a one-to-one correspondence between the theoretical (uniform) dose and the experimental effective dose.

**FIG. 3.**
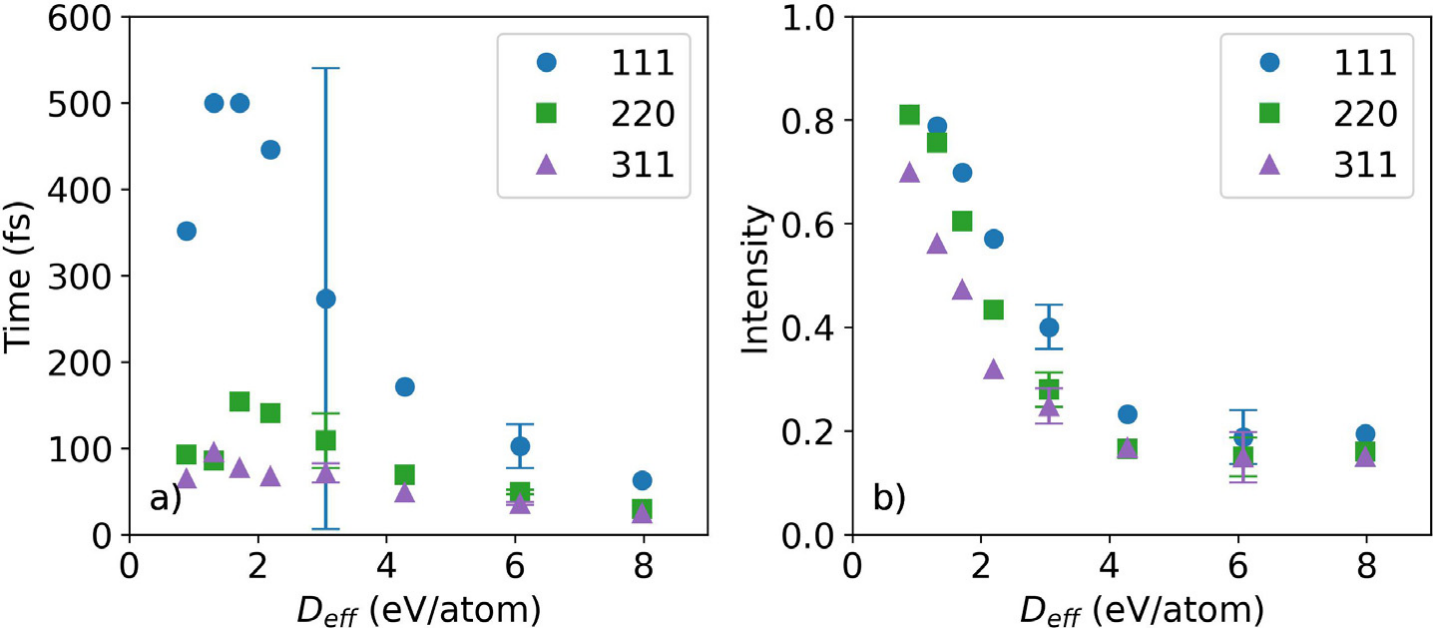
The (a) time constants and (b) intensities at 250 fs from the exponential fits of the (111), (220), and (311) diffraction intensity curves.

The curves in Fig. 2 represent fits with an exponential function. Figure 3 shows the time constants and the diffraction intensities at 250 fs from the exponential fits. In Fig. 3, selected error bars are shown. For each reflection and at each x-ray dose, the diffraction intensities decrease with time, while no new peaks were observed, as shown in Fig. 1(c). It is concluded that there is an ultrafast disordering of the crystal lattice, but the diamond symmetry is preserved. As expected, the diffraction intensity at the maximum time delay, 250 fs, decreases with increasing x-ray dose as the sample becomes more disordered. A general trend is observed where the time constant for the changing diffraction intensity becomes faster with the increasing x-ray dose. In addition, the (111) time constants are observed to be longer than those of the (220) and (311) reflections.

The decreasing diffraction intensities can be understood further from a Debye–Waller analysis. In this model, the diffraction intensity is reduced by exp $\left(-q^{2}\left\langle u^{2}\right\rangle \right)$, where q is the scattering vector, q = 4πsinθ/λ, 2θ is the scattering angle, λ is the x-ray wavelength, and $\sqrt{\left\langle u^{2}\right\rangle }$ is the root mean square (rms) atomic displacement perpendicular to a particular lattice plane. At zero pump-probe time delay, $\sqrt{\left\langle u_{0}^{\;2}\right\rangle }$= 0.043 Å corresponds to the rms displacement of carbon atoms in room temperature diamond.^34^ The Debye–Waller theory is valid for small atomic displacements.^35^ In the case of the ultrafast melting of silicon, it has been concluded that the Debye–Waller theory was correct for displacements $\sqrt{\left\langle u^{2}\right\rangle }$ up to ∼1 A,^36^ compensating for the relative dimensions of the diamond and silicon unit cells and for the projection of *u* onto a direction hkl gives $\sqrt{\left\langle u_{\text{hkl}}^{\;2}\right\rangle }$ ∼ 0.4 Å as an upper limit. An additional confirmation of the Debye–Waller analysis can be made by calculating the rms displacements directly from the XTANT+ simulation's atomic positions and by evaluating the rms displacements using the Debye–Waller model with the simulation's diffraction intensities.^37^ For all three x-ray doses 0.75, 2.50, and 3.88 eV/atom, the atomic displacements from the atomic positions and from the Debye–Waller model agree within 10%.

**FIG. 4.**
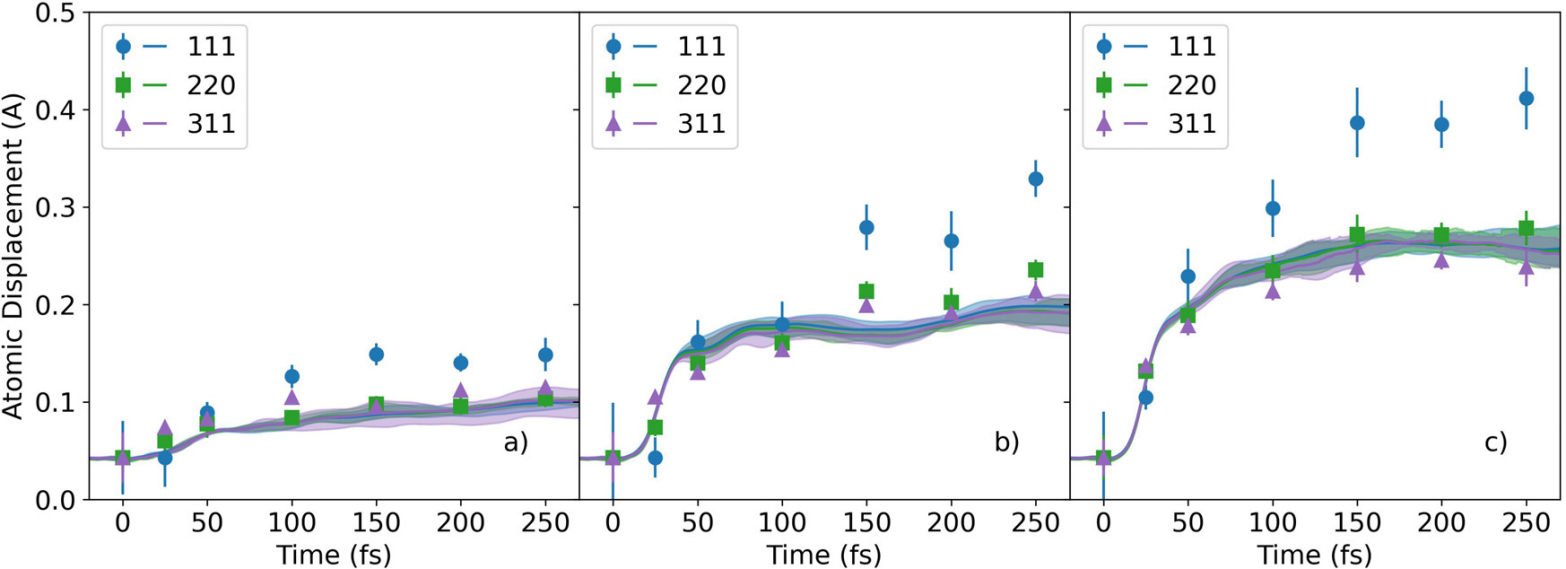
The time-dependent root mean square displacements calculated from the experimental (111), (220), and (311) diffraction peak intensities at the effective x-ray doses of (a) 0.9, (b) 3.1, and (c) 6.1 eV/atom and the root mean square displacements from the simulations at the x-ray doses of (a) 0.75, (b) 2.50, and (c) 3.88 eV/atom.

Figure 4 shows the experimental and simulated rms atomic displacements calculated for the three diffraction peaks. At early time delays, the experimental atomic displacements derived from the three reflections are the same within the uncertainties, which indicates that the motion is isotropic. At all the x-ray fluences and later time delays, the experimental atomic displacements perpendicular to the (111) planes are substantially higher than that perpendicular to the (220) and (311) planes. It is noted that the (111) direction is along the carbon–carbon bond, which is weakened by the excitation of electrons into the conduction band.^38^ The atomic displacements from the simulations, Figs. 4(a)–4(c), are discussed in Sec. IV.

**FIG. 5.**
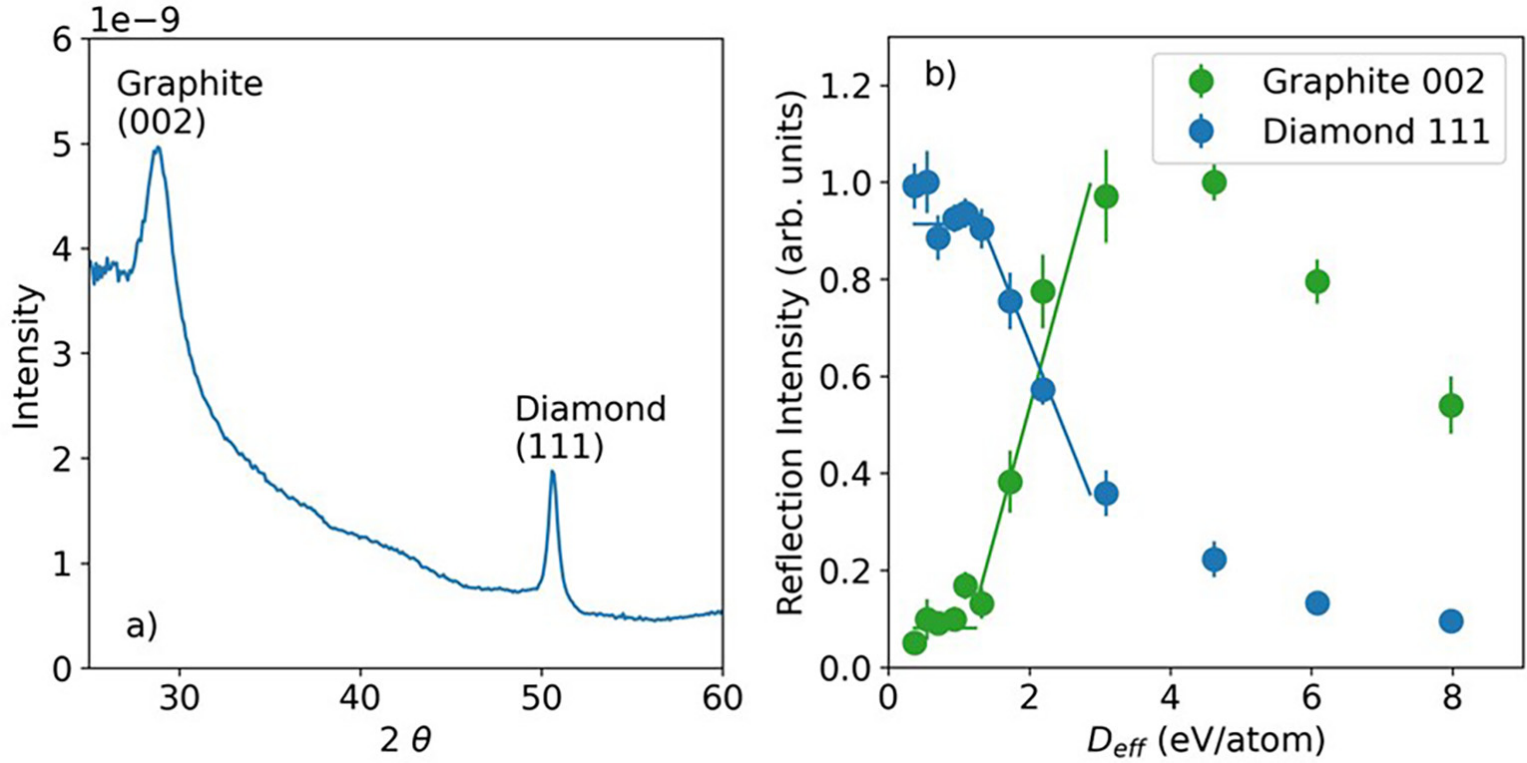
(a) The azimuthally integrated diffraction pattern and (b) the intensities of the diamond (111) and graphite (002) diffraction peaks measured at a delay of 33 ms.

At SACLA, x-ray pulses are delivered to beamline 3 at a repetition rate of 30 Hz. By accepting two pump pulses without probe pulses at individual sample locations, one measures the x-ray diffraction at a long time delay, 33 ms. Figure 5(a) shows the azimuthally integrated diffraction pattern and Fig. 5(b) the intensity of the diamond (111) and graphite (002) diffraction peaks with the maximum intensities normalized to 1. The sample was translated after each double x-ray pump exposure, with a sequential motion. There were between 401 and 546 exposures for each x-ray spot size. The graphite (002) peak appears at a 2θ of 29.1°, which is somewhat shifted from the nominal angle of 30.7°. The peak is also broad with a width of ∼2.3° (fwhm). Interestingly, shock compressed graphite-diamond mixtures display a broad graphite (002) peak at a 2θ angle equivalent to 29.0° at the pump photon energy, 7 keV.^39^ These observations suggest that the graphite crystals are affected by stress.

The observed threshold for graphitization was found to be at *D*_eff_ of 1.2 ± 0.1 eV/atom. The threshold was evaluated by fitting the graphite (002) and diamond (111) intensity data to constants and lines and then finding the intersection points between them, as shown in Fig. 5. The error bar is derived from the standard deviation of the fits. This threshold is similar to the non-thermal ones obtained from the tight-binding molecular dynamics (∼1.0 eV/atom with XTANT) and the density-functional tight-binding molecular dynamics (1.3 eV/atom with XTANT+) calculations.^8^ It exceeds the graphitization damage threshold observed experimentally by Gaudin *et al.*^6^ (∼0.7 eV/atom). The threshold from Gaudin *et al.* has the advantage that at XUV photon energies, the electron cascade size in diamond is in the nm range^20^ and does not have a significant influence on the x-ray dose. The long timescale required to observe the graphite diffraction peak implies that graphitization may occur by a thermal process, rather than non-thermal one.^6,7^

At a high x-ray dose, about 4 eV/atom and above, the intensity of the graphite (002) and diamond (111) peaks both decrease, indicating a regime of ablation or permanent structural disordering, similar to that predicted theoretically.^8^ In a limited postmortem analysis of the samples, scanning probe microscopy showed indentations 1 *μ*m wide and 50–100 nm deep, indicating that some ablation does occur.

## IV. SIMULATIONS AND DISCUSSION

**FIG. 6.**
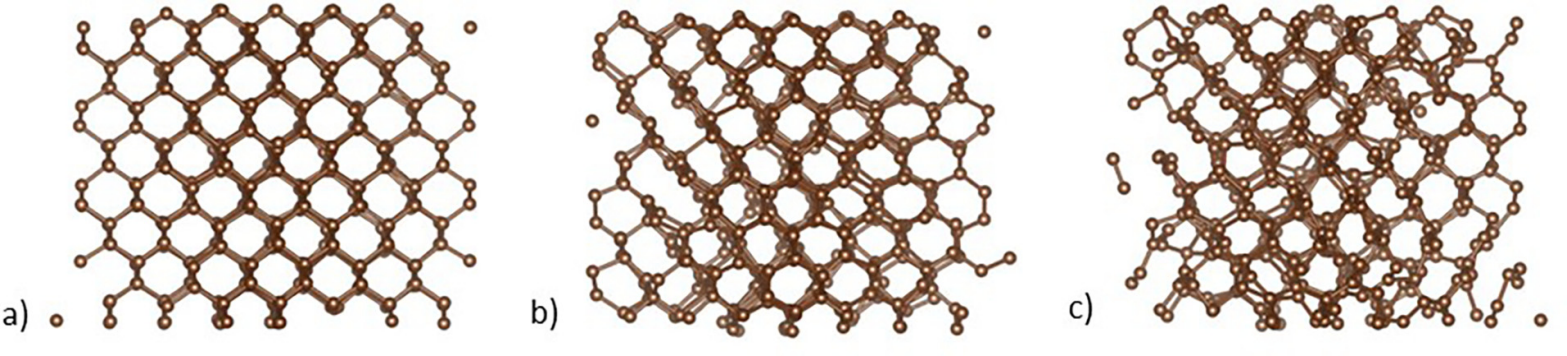
XTANT+ atomic snapshots at x-ray doses of (a) 0.75, (b) 2.5, and (c) 3.88 eV/atom and time delay of 250 fs.

Figure 4 displays the root mean square atomic displacements from XTANT+ simulations and Fig. 6 the corresponding atomic snapshots. The XTANT+ calculations were performed with 512 atoms in a simulation box with periodic boundaries, at 0.05 fs molecular dynamics time step, and with 100 000 Monte Carlo iterations. Five molecular dynamics realizations were performed at each x-ray dose. The application of averaged electron distributions removes strong fluctuations from the molecular dynamics. Figure 4 shows the rms atomic displacements from the simulations at three x-ray doses: 0.75, 2.5, and 3.88 eV/atom with homogeneous x-ray absorption. The simulations were performed at x-ray doses similar to the effective x-ray doses used in the experiment. In the calculations, all the atoms experience a constant x-ray dose. In contrast, in the experiment, because of the Gaussian profiles of the pump and probe beams, the sample is excited by a range of x-ray doses. The difficulty of comparing simulations with x-ray pump-probe experiments has been discussed in depth in Ref. 40. In Fig. 4, a measure of simulation error is given as bands from the minimum to maximum rms atomic displacement chosen from all the MD realizations performed. They are plotted together with curves showing the rms displacements averaged over the number of MD realizations.

In the XTANT+ atomic displacements shown in Fig. 4, dynamics are observed on several time scales. The atomic displacements remain nearly constant until ∼10 fs after the pump pulse similar to what was previously observed by Inoue *et al.*^5,11^ The delayed onset for atomic disordering is not observable in the experiment because of the lack of sufficient data points at short time delays. Then, the atomic displacements increase sharply until ∼40 fs. Both the delayed onset and the fast increase in the atomic displacements occur during the timescale of the electron cascading, during which the collisions of photoelectrons and Auger electrons with atoms result in excitations of the valence electrons to the conduction band. Then, a slower increase in the atomic displacements occurs. This behavior at later times takes place after the electron cascading is finished, and the atoms move in the new potential energy surface. In Fig. 4, good agreement is found between the experimental and simulation results for the atomic displacements in the (220) and (311) directions. The experimentally observed difference between the atomic displacements in the (111) direction compared with those in the (220) or (311) directions is not found in the XTANT+ simulation results. While performing a volume integration of simulations at different x-ray doses according to the x-ray pump and probe profiles would improve the quantitative agreement between the experiment and simulations, it was outside the scope of the present study because of the required computer resources. Overall, the simulations in Fig. 4 provide qualitative agreement with the experimental results in terms of time scales and trends.

Figure 6 shows XTANT+ atomic snapshots from individual molecular dynamics realizations. This figure has been generated using VESTA 3.^41^ The disorder is seen to increase with the x-ray dose. At 2.5 eV/atom, evidence of graphite planes is observed in the upper left of the simulation box, although in the other molecular dynamics realizations, the graphite planes were less clearly seen. For the short-range order, carbon pair distribution functions predicted by XTANT+^8^ show a well-defined peak for the second nearest-neighbor at an x-ray dose of 1.0 eV/atom. At 3.0 eV/atom, there is only a well-defined peak for the first nearest-neighbor.

The experiment does not observe extra diffraction peaks from graphite on the femtosecond timescale although the graphite (002) reflection is detected at the pump-probe time delay of 33 ms. From the XTANT+ atomic structures using the XSINC code,^37^ one may derive diffraction intensities for particular reflections including graphite (002). The graphite peak is orders of magnitude lower than the diamond peaks. In addition, in Fig. 6, the graphite structure is seen over a fraction of the simulated atoms indicating small graphite domains, which would result in broad diffraction peaks. These weak broad diffraction peaks would not be observable in the experiment. The experiment provides evidence for disordering on an ultrafast timescale and graphitization on a long timescale, which may be of thermal origin. The simulations show partial graphitization but predominantly disordering on the ultrafast timescale.

## Data Availability

The data that support the findings of this study are available from the corresponding authors upon reasonable request.
